# Viral Bone Infection: A Neglected Disease?

**DOI:** 10.3390/microorganisms8060797

**Published:** 2020-05-26

**Authors:** Lorenzo Drago, Carlo L. Romanò, Ilaria Morelli, Thami Benzakour

**Affiliations:** 1 Clinical Microbiology, University of Milan, 20100 Milano, Italy; 2 Studio Medico Cecca-Romanò, Corso Venezia, 20121 Milano, Italy; carlo.romano@unimi.it; 3 Romano Institute, Rruga Ibrahim Rugova 1, 00100 Tirane, Albania; 4 Orthopedics Specialty School, University of Milan, 20100 Milano, Italy; ilaria.morelli@unimi.it; 5 Zerktouni Orthopaedic Clinic, 20000 Casablanca, Morocco; t.benzakour@gmail.com

## Abstract

Bone structures reveal viral DNA/RNA, but little is known of the interaction and pathogenesis of viruses and bone diseases. Their detection and identification is often overlooked and not considered by many clinicians and researchers. In this Editorial, we suggest the role of viruses in some inflammatory bone conditions and their possible role as aetiological agents in bone and joint infections.

In recent decades, orthopaedic surgeons, infectious disease specialists, and microbiologists have been nearly exclusively focused on bone infections due to bacteria and, much more rarely, fungi [1,2,3]. Fungi and bacteria are routinely tested in the laboratories in cases of suspected infections by applying cultural and (sometimes) molecular methods, while viruses are never searched because it is believed that these microorganisms cannot cause a bone infection.

Although viruses are ubiquitous and viral infections have been described for virtually all human organs and tissues, viral osteomyelitis is still a somewhat mysterious entity, whose clinical impact seems negligible.

The reason for the different impact between bacteria/fungi and viruses may be attributable to the techniques routinely used: traditional cultural methods for the former, which cannot be always used instead for the viruses. The advent of molecular methods has increased the diagnostic reliability of viruses at accessible costs. Nevertheless, viruses do not appear to be of interest in the orthopedic field. It has been said that “bones hold the key to DNA virus history and epidemiology” [4]; however, little is known about the interaction of viral particles and bone and how this may eventually contribute to the occurrence of pathological conditions. 

Certainly, bony sequelae of viral infections have been reported in the past, as for “osteomyelitis *variolosa*” in smallpox, associated with several kinds of bone deformities, like short stubby metacarpals and phalanges, joint ankylosis, joint multidirectional instability, and hypoplastic femoral condyles [5]. However, evidence of viral osteomyelitis per se is a relatively new finding.

Similarly, the literature is quite rich concerning viral arthritis pathogenesis. In fact, several viruses have been recognized as aetiological agents of viral arthritis, including Parvovirus B19, Hepatitis A (HAV), B (HBV) and C virus (HCV), Rubella virus, Alphaviruses, Flaviviruses, and Retroviruses, with HAV and HBV accounting for approximately 10–14% and 20–25% of all viral arthritides, respectively [6]. However, viral arthritis is often explained by virus-induced autoimmunity and resulting inflammatory response, rather than the result of direct infection with primary damage to cartilage tissues. 

The advent of new genomic and sequencing technologies has uncovered in recent years a myriad of viral sequences that were previously unknown to coexist with humans. For instance, human parvovirus B19 (B19V), a highly prevalent DNA virus, has been detected in a range of tissues and organs, including bone. This virus has been shown to replicate in erythroid progenitor cells in the bone marrow and in mesenchymal stromal cells, which can differentiate into cartilage and bone [4].

Some viruses have been recently found able to effectively interact with bones. 

Transfection of normal human osteoclast precursors with the Measles virus (MV) nucleocapsid or Measles virus infection of bone marrow cells from transgenic mice expressing a MV receptor has been shown to result in formation of pagetic-like osteoclasts [7].

Countries such as New Zealand, Vietnam, Laos, and Cambodia know the concerns raised by Zika virus infections. Although Zika pathogenesis and bone-related pathology remain unknown, this virus induces arthralgia due to perturbed osteoblast function [8].

An indirect and direct role of human immunodeficiency viruses (HIV) on bone infections has been also evidenced by the medical literature. A recent paper provides results emphasizing the significantly elevated risk of periprosthetic joint infections in HIV-positive patients [9]. Even more interestingly, HIV-1 has been shown to directly infect osteoclasts, causing bone resorption and bone defects. In particular, HIV-1 seems to contribute to enhanced bone degradation in human osteoclasts by modifying the structure and function of the sealing zone, the bone resorption machinery of osteoclasts [10].

Alphaviruses have also been reported to interfere with bone remodelling. In particular, Ross River virus (RRV), a small, single-stranded RNA virus, transmitted by mosquitoes, causes bone resorption by the direct infection of osteoblasts [11]. Other Alphaviruses, including Chikungunya virus (CHIKV), Sindbis virus (SINV), o’nyong-nyong virus (ONNV), Mayaro virus, and Barmah Forest virus (BFV), all feature variable degree of arthritis with bone and joint lesions. 

Of note, bone loss caused by Alphavirus infection can be prevented by inhibition of interleukin-6 (IL-6). Alphaviruses may disrupt bone homeostasis by upregulating IL-6, which contributes to bone loss by disrupting the receptor activator of nuclear factor-kappaB ligand/osteoprotegerin balance [11].

Cytomegalovirus and other human herpes viruses, as well as other viruses, have been found in bones, joints or in the synovial fluid, causing “non-specific inflammation”. Furthermore, acute skull osteomyelitis after Varicella–Zoster Virus (VZV) reactivation and Propionibacterium acnes coinfection has been described [12].

Herpes simplex virus (HSV) and cytomegalovirus (CMV) are also indicated as rare causes of arthritis after bone marrow transplantation.

Despite the fact their direct involvement in bone infections has never been demonstrated, other viruses capable of causing arthritis deserve attention during the differential diagnosis. 

Epstein–Barr virus is usually associated with polyarthralgia, or monoarthritis of the knee. VZV can be involved in children pauciarticular arthritis [13]. Mumps virus can be associated with small or large joint synovitis. Adenovirus or Coxsackieviruses A9, B2, B3, B4, and B6 have been associated with recurrent episodes of polyarthritis as well as Echoviruses.

The role of viruses in the orthopaedic field has been neglected so far. Although the interaction of viruses with bone tissue has been poorly investigated in recent decades, as far as we know they exert bone pathogenicity via several mechanisms, including direct synovial infection leading to cell lysis, immune complex formation, or proinflammatory cytokines induction. 

This editorial raises the question of whether the connection between viruses and bone infections really exists or is just a casuality. There is much evidence supporting this relationship, so understanding if it is a neglected disease or not should be mandatory for the scientific community.

We need to look more deeply at these new etiological agents in the future.

This editorial aims to increase awareness in the orthopedic field as well as in microbiology in order to reconsider this topic by looking at new methodological approaches. Therefore, the matter represents an urgent need from methodological, clinical, and procedural points of view.
